# Serial analysis of gene expression identifies putative metastasis-associated transcripts in colon tumour cellines

**DOI:** 10.1054/bjoc.2000.1330

**Published:** 2000-08-17

**Authors:** A Parle-McDermott, P McWilliam, O Tighe, D Dunican, D T Croke

**Affiliations:** Department of Biochemistry, Royal College of Surgeons in Ireland, 123 St Stephen’s Green, Dublin 2, Ireland

**Keywords:** SAGE, colon, differential, metastasis, expression

## Abstract

We have used serial analysis of gene expression (SAGE) to identify gene expression differences between a primary colon tumour cell line (SW480) and an isogenic lymph-node metastasis cell line (SW620). Differential expression was confirmed for the following genes: keratin K5, cystatin S, serum amyloid A, the human homologue of yeast ribosomal S28 and the p32 subunit of human pre-mRNA splicing factor SF2. Expression of confirmed differences were also analysed in other metastatic cell lines. © 2000 Cancer Research Campaign


British Journal of Cancer (2000) 83(6), 725–728
© 2000 Cancer Research Campaign
doi: 10.1054/ bjoc.2000.1330, available online at http://www.idealibrary.com on IDEAL®

### Serial analysis of gene expression identifies putative metastasis-associated transcripts in colon tumour cell lines

**A Parle-McDermott, P McWilliam, O Tighe, D Dunican and DT Croke**

Department of Biochemistry, Royal College of Surgeons in Ireland, 123 St Stephen's Green, Dublin 2, Ireland

**Summary** We have used serial analysis of gene expression (SAGE) to identify gene expression differences between a primary colon tumour cell line (SW480) and an isogenic lymph-node metastasis cell line (SW620). Differential expression was confirmed for the following genes: keratin K5, cystatin S, serum amyloid A, the human homologue of yeast ribosomal S28 and the p32 subunit of human pre-mRNA splicing factor SF2. Expression of confirmed differences were also analysed in other metastatic cell lines. © 2000 Cancer Research Campaign

**Keywords**: SAGE; colon; differential; metastasis; expression

We have used the method of serial analysis of gene expression (SAGE) (Velculescu et al, 1995) to identify changes in gene expression during colorectal cancer progression. Although SAGE has already been applied to an analysis of colorectal cancer (Zhang et al, 1997), that study focused on gene expression differences between normal and tumour tissues/cell lines. Our study focused on identifying expression differences among highly expressed genes between a primary colorectal tumour cell line (SW480) and an isogenic lymph-node metastasis cell line (SW620) isolated from the same patient (Leibovitz et al, 1976), thus providing information on the gene expression changes which tumour cells may undergo in order to become metastatic.

### MATERIALS AND METHODS

### Cell culture

The cell lines SW480, SW620, SW1116, LoVo, Colo201, T84 (ATCC, USA) and 498LI (Lieberman et al, 1991) were cultured under conditions recommended by the supplier.

### Serial analysis of gene expression (SAGE)

SAGE was carried out on the isogenic cell lines SW480 and SW620 essentially as previously described (Velculescu et al, 1997; Zhang et al, 1997). Clones containing at least 10–20 tags were sequenced and analysed on an ALFexpress™ automated sequencer (Pharmacia, UK). Sequence files were analysed using the SAGE software version 1.0 (Velculescu et al, 1995).

### Confirmation of differential expression

Semi-quantitative RT-PCR amplifications were based on the method of He et al (1995) with modifications including a Cy5-labelled primer in all PCR amplifications. Gene-specific primer sequences are available from the authors upon request. Cy5-labelled PCR products were analysed on an ALFexpress™ automated sequencer (Pharmacia). Northern blotting was carried out by resolving total RNA on a 1% denaturing formaldehyde gel and transferring onto Hybond N$^+$ (Amersham, UK) nylon membrane by capillary blotting. Transcript sizes were estimated by migration relative to 28S and 18S ribosomal RNA. Sequential hybridizations were carried out overnight at 65°C with purified cDNA fragments radiolabelled with [$\alpha$-$^{32}$P] dCTP by the random prime method using the Rediprime® labelling kit II (Amersham, UK).

*Received 20 October 1999*
*Revised 11 April 2000*
*Accepted 11 May 2000*

*Correspondence to:* DT Croke

### RESULTS

### Generation and analysis of SAGE libraries

SAGE was applied to the cell lines SW480 and SW620 as candidate pre-metastatic and metastatic cell populations. The total number of 'tags' sequenced included 5079 tags from SW480 and 5044 from SW620. Comparison of tag abundances between the two libraries showed that the majority of genes were expressed at comparable levels between the two. As the number of tags sequenced only numbered approximately 5000 from each cell line, the significance of tag abundance differences had to be confirmed by an independent method. We examined the expression of ten putative differentially expressed genes identified from the SAGE data (Table 1); only half were confirmed as true differences between SW480 and SW620 by semi-quantitative RT-PCR or Northern blot.

### Analysis of expression differences by semi-quantitative RT-PCR and Northern blotting

Analysis of the expression of myosin light-chain, high-mobility group box protein (SSRP1), β-tubulin, hnRNP L and hnRNP C by semi-quantitative RT-PCR revealed no significant differences in expression between SW480 and SW620 (data not shown) and thus were not analysed further. The human homologue of yeast

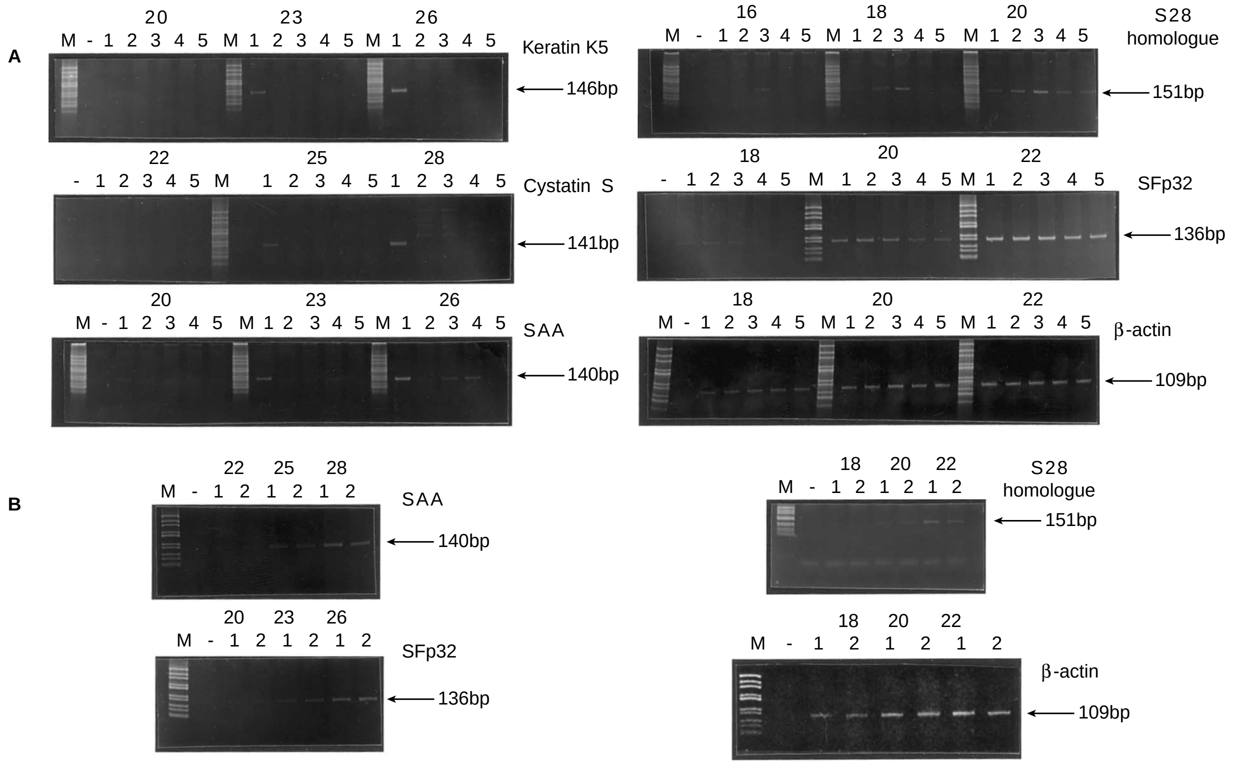

**Figure 1** Semi-quantitative RT-PCR products resolved by electrophoresis on 12% polyacrylamide gels. M: molecular weight marker; – : negative control. The numbers written in bold refer to the cycle numbers sampled for each PCR amplification. β-actin was amplified in parallel as a control. (**A**) 1: SW480, 2: SW620, 3: LoVo, 4: Colo201, 5: T84. (**B**) 1: SW1116, 2: 498LI. Note: keratin K5 and cystatin S were not detected in SW1116 or 498LI and thus are not shown here.

ribosomal S28, the p32 subunit of human pre-mRNA splicing factor SF2, keratin type II K5, cystatin S and serum amyloid A (SAA) all showed differential expression in the metastatic cell line (SW620) relative to the primary tumour cell line (SW480) (Figure 1A, lanes 1 and 2) (Table 2). The cDNA products generated from the RT-PCR analysis were purified and used for Northern blot analysis (Figure 2). All five cDNA probes hybridized to transcripts of the expected size and differential expression was also further confirmed.

### Analysis of expression in other cell lines

We examined the level of expression of the five confirmed differentially expressed genes in other colon carcinoma cell lines by semi-quantitative RT-PCR. These included a pair of cell lines: SW1116, a human colon adenocarcinoma line and 498LI (Lieberman et al, 1991), a metastatic variant of SW1116 selected in vitro (Figure 1B, lanes 1 and 2) (Table 2). Three metastatic cell lines, LoVo, Colo201 and T84, all originating from primary colon tumours, were also examined (Figure 1A, lanes 3–5). The expression data is summarized in Table 2.

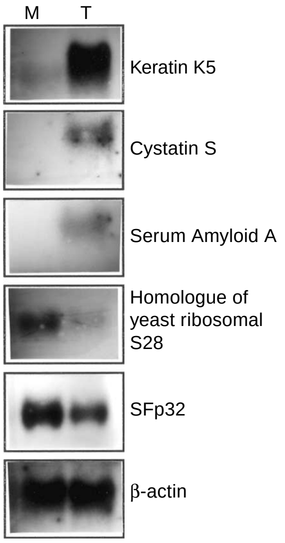

**Figure 2** Northern blot analysis of the confirmed differentially expressed genes identified by SAGE. T refers to the primary tumour cell line SW480 and M refers to the metastatic cell line SW620. The blot was probed with β-actin to control for equal loading and transfer.

### DISCUSSION

Our SAGE analysis has allowed a comparison between highly expressed genes and, thus far, we have further examined the expression of ten genes showing differential expression. We have found that a SAGE analysis based on approximately 5000 tags per cell type results in a number of false-positives, despite the 'house-keeping' genes GAPDH and β-actin (Cleveland et al, 1980; Fort et al, 1985) showing a similar tag number (Table 1). All of the false-positives that we identified were represented by <10 tags in

 *© 2000 Cancer Research Campaign*

**Table 1** Subset of differentially expressed tags further analysed

| SAGE Tag | T/M | Gene | Accession Number |
|---|---|---|---|
| CTGTTGGTG | 1/17 | **Human homologue of yeast ribosomal S28** | D14530 |
| GCCCCTGCT | 18/1 | **Keratin type II K5** | M21389 |
| GTACACACA | 15/1 | **Cystatin S** | X54667 |
| GTGCTGAAT | 8/1 | Myosin light-chain mRNA | U02629 |
| GTGCGGAGG | 7/0 | **Serum amyloid A** | X51439 |
| TACTCTTGG | 0/7 | hnRNPL | X16135 |
| TGAGGCCAG | 6/0 | High-mobility group box (SSRP1) mRNA | M86737 |
| ATAGACATA | 1/6 | **p32 subunit of Splicing Factor 2** | M69039 |
| AACGACCTC | 1/6 | β-tubulin | V00599 |
| TCAAATGCA | 0/5 | hnRNP C | M16342 |
| *TACCATCAA | 32/26 | Glyceraldehyde 3-phosphate dehydrogenase | J02642 |
| *GCTTTATTT | 9/8 | β-actin | X00351 |

T/M is the ratio of tags from the primary tumour cell line SW480 (T) compared to the metastatic cell line SW620 (M). Genes written in **bold** were confirmed by independent methods (see text). *The 'housekeeping' genes GAPDH and β-actin showed a similar tag abundance as expected

**Table 2** Fold differences in expression between primary tumour and metastatic cell lines

| Gene transcript | SW480 | **SW620** | **LoVo** | **Colo201** | **T84** | SW1116 | **498LI** |
|---|---|---|---|---|---|---|---|
| **Fold decreased expression in metastatic cell lines**[a] | | | | | | | |
| Keratin type II K5 | 1.0 | nd | nd | nd | nd | nd | nd |
| Cystatin S | 1.0 | nd | nd | nd | nd | nd | nd |
| Serum amyloid A | 1.0 | nd | 16 | 8.6 | nd | 1.0 | 1.4 |
| **Fold increased expression in metastatic cell lines**[b] | | | | | | | |
| p32 subunit of splicing factor 2 | 1.0 | 2.0 | 1.6 | 0.8 | 0.7 | 1.0 | 2.2 |
| Human homologue of yeast ribosomal S28 | 1.0 | 2.2 | 6.2 | 0.9 | 0.6 | 1.0 | 0.7 |

Cell lines written in **bold** are metastatic and those written in roman are the primary colon tumour cell lines. Fold differences were calculated based on β-actin ratios and relative to expression in primary tumour cell lines. The results are based on repeated experiments with an overall average standard error of the mean of 4.1%. Cell lines SW620, LoVo, Colo201 and T84 are described relative to SW480, and 498LI is described relative to SW1116; nd = not detected; [a]Fold differences are described as decreased expression in the metastatic cell lines relative to the primary tumour cell lines; [b]Fold differences are described as increased expression in the metastatic cell lines relative to the primary tumour cell lines abundance. Therefore, increasing the number of tags sequenced is likely to reduce or eliminate the number of false-positives.

We have confirmed differential expression of five genes between SW480 and SW620 (Table 2). Cystatin S and keratin type II K5 were not expressed at a significant level in the four other metastatic cell lines that we examined (LoVo, Colo201, T84 and 498LI) or in the primary tumour cell line SW1116. Cystatin S is a member of the cysteine proteinase inhibitor superfamily (Bobek et al, 1991) and other members, such as cystatin M, have been previously associated with metastatic spread (Sotiropoulou et al, 1997). Keratin K5 is a type II cytoskeletal intermediate filament protein (Fuchs and Weber, 1994) and the cytoskeletal structures are known to play a major role in cell motility and invasion, proliferation, differentiation and in the transduction of extracellular signals (Sherbet and Lakshmi, 1997).

Serum amyloid A (SAA) showed varying degrees of loss of expression in all of the metastatic cell lines that we examined. SAA is an acute-phase reactant and is found in the circulation as an apolipoprotein bound to HDL (high density lipoprotein) (apoSAA) (Strachan et al, 1989). The potential role that SAA may have in metastasis is currently unclear but the observed increase in expression of SAA in response to p53-induced apoptosis (Polyak et al, 1997) may be significant.

We also observed increased expression of the p32 subunit precursor of pre-mRNA splicing factor SF2, and the human homologue of yeast ribosomal S28 in the metastatic cell line(s) relative to their primary tumour cell line counterpart(s) (Table 2). The interaction of p32 with the pre-mRNA splicing factor SF2 (Krainer et al, 1991; Honore et al, 1993) and its ability to bind hyaluronic acid (HA) (Deb and Datta, 1996) suggests a potential role in metastasis. Increased expression of the human homologue of yeast ribosomal S28 has also been found in colon tumours as compared to normal tissue (Zhang et al, 1997). This indicates that increased expression of S28 may be correlated with colon cancer progression.

In conclusion, we have shown here that analysis of a moderate number of SAGE tags (approximately 10 000 in total) has allowed us to identify a number of differentially expressed genes that may be involved in colon tumour metastasis. However, our analysis has also shown that, with a moderate level of SAGE tags, comparison of the gene expression profiles must be interpreted with caution as tag abundances <10 do not consistently represent true differences in expression. This agrees with another recent SAGE analysis of endothelial cells which consisted of over 12 000 tags (de Waard et al, 1999). The five differentially expressed genes that we have confirmed have not been implicated in colon tumour progression

*© 2000 Cancer Research Campaign*

previously and may contribute to our understanding of metastasis. However, it is clear that our study requires further analysis in order to show a direct association between the identified differentially expressed genes and the metastatic phenotype.

### ACKNOWLEDGEMENTS

We wish to thank Drs Jill Powell (ICRF, UK) and Victor Veculescu (Johns Hopkins, MD, USA) for helpful advice in implementing the SAGE protocol. We also thank Dr Noel Williams (PA, USA) for his continued interest and support of the project and Dr Meenhard Herlyn (Wistar Institute, PA, USA) for the kind gift of 498LI. This work was funded by the Health Research Board of Ireland.

### REFERENCES

Bobek LA, Aguirre A and Levine MJ (1991) Human salivary cystatin S. *Biochem J* **278**: 627–635

Cleveland DW, Lopata MA, MacDonald RJ, Cowan NJ, Rutter WJ and Kirschner MW (1980) Number and evolutionary conservation of α- and β-tubulin and cytoplasmic β- and γ-actin genes using specific cloned cDNA probes. *Cell* **20**: 95

Deb TB and Datta K (1996) Molecular cloning of human fibroblast Hyaluronic Acid-binding protein confirms its identity with P-32, a protein co-purified with splicing factor SF2. *J Biol Chem* **271**: 2206–2212

de Waard V, van den Berg BMM, Veken J, Schultz-Heienbrok R, Pannekoek H and van Zonneveld A (1999) Serial analysis of gene expression to assess the endothelial cell response to an atherogenic stimulus. *Gene* **226**: 1–8

Fort P, Marty L, Piechaczyk M, el Sabrouty S, Dani C, Jeanteur P and Blanchard JM (1985) Various rat adult tissues express only one major mRNA species from the glyceraldehyde-3-phosphate-dehydrogenase multigenic family. *Nucleic Acids Res* **13**: 5

Fuchs E and Weber K (1994) Intermediate filaments: structure, dynamics, function, and disease. *Annu Rev Biochem* **63**: 345–382

He M, Liu E and Conway K (1995) Semi-quantitative reverse transcriptase-polymerase chain reaction for the detection of low abundance transcripts in human cells. In *Reverse Transcriptase PCR*, Larrick JW and Siebert PD (eds) pp 289–299. Ellis Horwood: London

Honore B, Madsen P, Rasmussen HH, Vandekerckhove J, Celis JE and Leffers H (1993) Cloning and expression of a cDNA covering the complete coding region of the P32 subunit of human pre-mRNA splicing factor SF2. *Gene* **134**: 283–287

Krainer AR, Mayeda A, Kozak D and Binns G (1991) Functional expression of cloned human splicing factor SF2: homology to RNA-binding proteins, U1 70K, and *Drosophila* splicing regulators. *Cell* **66**: 383–394

Leibovitz A, Stinson JC, McCombs WB, McCoy CE, Mazur KC and Mabry ND (1976) Classification of human colorectal adenocarcinoma cell lines. *Cancer Res* **36**: 4562–4569

Lieberman MD, Sigal RK, Williams NN and Daly JM (1991) Natural killer cell stimulatory factor (NKSF) augments natural killer cell and antibody-dependent tumoricidal response against colon carcinoma cell lines. *J Surg Res* **50**: 410–415

Polyak K, Xia Y, Zweier JL, Kinzler KW and Vogelstein B (1997) A model for p53-induced apoptosis. *Nature* **389**: 300–305

Sherbet GV and Lakshmi MS (1997) Dominant metastasis-associated genes. In *The Genetics of Cancer: genes associated with cancer invasion, metastasis, and cell proliferation*, pp 190–213. Academic Press London

Sotiropoulou G, Anisowicz A and Sager R (1997) Identification, cloning and characterization of cystatin M, a novel cysteine proteinase inhibitor, down-regulated in breast cancer. *J Biol Chem* **272**: 903–910

Strachan AF, Shephard EG, Bellstedt DU, Coetzee GA, van der Westhuyzen DR and de Beer FC (1989) Human serum amyloid A protein. *Biochem J* **263**: 365–370

Velculescu VE, Zhang L, Vogelstein B and Kinzler KW (1995) Serial analysis of gene expression. *Science* **270**: 484–487

Velculescu VE, Zhang L, Zhou W, Vogelstein J, Basrai MA, Bassett DE Jr, Hieter P, Vogelstein B and Kinzler KW (1997) Characterization of the yeast transcriptome. *Cell* **88**: 243–251

Zhang L, Zhou W, Velculescu VE, Kern SE, Hruban RH, Hamilton SR, Vogelstein B and Kinzler KW (1997) Gene expression profiles in normal and cancer cells. *Science* **276**: 1268–1272

*© 2000 Cancer Research Campaign*
